
JOURNAL INFORMATION
==============================
NLM Title Abbreviation: South Med Rec
NLM Journal ID: 0027317

The Southern Medical Record

Publisher: Biomedical Journal Digitization Project

ARTICLE INFORMATION
==============================
PMCID: PMC9084166
PMID: 36022476
Article version: 1
Subjects: Editorial and Miscellaneous

Medical Association of Alabama

Print publication date: 1875 Aug
Volume: 5
Issue: 8
First page: 512
Last page: 512
License: This work is free of known copyright restrictions. For more information, please see PMC Back Issue Digitization.
License URL: https://creativecommons.org/publicdomain/mark/1.0/

==============================

*Medical Association of Alabama.*

We have just been presented by our esteemed friend and co-laborer, Dr. S. M. Hogan, with a copy of the "Transactions of the State of Alabama, held in the City of Montgomery in April last." It is a book of 360 pages, containing many valuable and instructive articles, some of which we hope to be able to give to our readers in the future. As heretofore, the proceedings honor the profession of the State. P.

### ADVERTISING RATES.

| | |
|---|---|
| For One Page, per annum | $60 00 |
| For Half Page, per annum | 30 00 |
| For Quarter Page, per annum | 16 00 |
| For One Page, one insertion | 10 00 |
| For Half Page, one insertion | 6 00 |
| For Quarter Page, one insertion | 4 |

A two-page printed Circular, mailed to us free of cost, will be inserted at the rate of Seven Dollars per issue. A four-page printed Circular, folded, will be inserted for Nine Dollars per issue.

Advertisers engaging space by the year, have the privilege of changing their advertisements for the cost of new composition. All advertisements will receive liberal Editorial notices.
